# Margins to compensate for respiratory-induced mismatches between lung tumor and fiducial marker positions using four-dimensional computed tomography

**DOI:** 10.1016/j.phro.2025.100728

**Published:** 2025-02-07

**Authors:** Seiya Matsumoto, Nobutaka Mukumoto, Tomohiro Ono, Hiraku Iramina, Hideaki Hirashima, Takanori Adachi, Yuki Miyabe, Noriko Kishi, Takashi Mizowaki, Mitsuhiro Nakamura

**Affiliations:** a Department of Advanced Medical Physics, Graduate School of Medicine, Kyoto University, 53 Kawahara-cho, Shogoin, Sakyo-ku, Kyoto 606-8507 Japan; b Department of Radiation Oncology, Graduate School of Medicine, Osaka Metropolitan University, 1-5-7 Asahi-machi, Abeno-ku, Osaka 545-0051 Japan; c Department of Radiation Oncology, Shiga General Hospital, 5-4-30 Moriyama, Moriyama, Shiga 524-0022, Japan; d Department of Radiation Oncology and Image-Applied Therapy, Graduate School of Medicine, Kyoto University, 54 Kawahara-cho, Shogoin, Sakyo-ku, Kyoto 606-8507, Japan; e Department of Radiation Oncology, Kitano Hospital, 2-4-20 Ogi-machi, Kita-ku, Osaka 530-8480, Japan

**Keywords:** Target localization error, Fiducial markers, Four-dimensional computed tomography, Stereotactic body radiotherapy, Lung

## Abstract

**Background and purpose:**

Tumors and fiducial markers do not always exhibit synchronous motion across different respiratory phases, in a phenomenon called the target localization error (TLE). We determined the margin to compensate for the TLE using four-dimensional computed tomography (4D-CT).

**Materials and methods:**

We analyzed data from 21 lung tumor patients with fiducial markers; 11 for TLE determination and 10 for validation. Shifted CT images were generated by aligning the centroids of the fiducial markers in the reference phase of 4D-CT with those in each respiratory phase, and the union of gross tumor volumes (GTVs) was determined (${GTV}_{union}^{shift}$). Conversely, variations in GTV centroids across the respiratory phases were calculated, and the 95th percentile of the root mean square error was defined as the TLE. Using this TLE, a GTV with an added TLE (${GTV}_{TLE}^{ref}$) was generated in the reference phase. Subsequently, a treatment plan assuming dynamic tumor tracking (DTT) was created for the planning target volume, derived by adding an isotropic 5 mm margin to ${GTV}_{TLE}^{ref}$, and the dose coverage for ${GTV}_{union}^{shift}$ was evaluated.

**Results:**

The TLEs (standard deviations of the root mean square error) were 2.0 (0.8) mm, 2.1(0.7) mm, and 3.2 (1.1) mm in the left − right, anterior − posterior, and superior − inferior directions, respectively. A dosimetric evaluation revealed that ${GTV}_{union}^{shift}$ did not receive 100 % of the prescribed dose in four of 10 cases owing to artifacts.

**Conclusion:**

The TLE can be compensated by adding an anisotropic margin to the GTV in the reference phase, a critical consideration in DTT.

## Highlights

• Twenty-one lung cancer patients with fiducial markers were analyzed.
• Localization errors in dynamic tumor tracking were determined.
• Localization errors were found to be 2.0, 2.1, and 3.2 mm in three directions.
• Four of 10 cases showed insufficient dose coverage due to severe motion artifacts.

## 1 Introduction

Stereotactic body radiotherapy (SBRT) is an effective treatment option, particularly for patients with early-stage non-small cell lung cancer (NSCLC) who are medically inoperable [1], [2]. Respiratory motion is a key consideration in SBRT for early-stage NSCLC. Depending on their anatomical location, lung tumors can exhibit movement exceeding 10 mm [1]. Moreover, amplitude variations and baseline drifts can occur [3], and the extent of respiratory motion may vary throughout the treatment course [4]. When setting the target volume considering these factors, the risk of adverse events can increase owing to the delivery of high doses over a wide area of the organs surrounding the tumor. The landmark QUANTEC paper reported that the incidence of adverse events increases as the mean lung dose and lung volume receiving radiation increase [5].

Therefore, implementing respiratory motion management strategies is a common practice for reducing the irradiated volume and minimizing the dose to organs at risk. Specific strategies for managing respiratory motion include breath-holding, respiratory gating, and dynamic tumor tracking (DTT) techniques [1]. The breath-holding technique involves the patient holding their breath to stabilize the tumor position during irradiation; however, it burdens the patient and requires high reproducibility of breath-holding. The respiratory-gating technique delivers radiation at a precise time when the respiratory phase matches the phase set during treatment planning; however, it requires waiting until the specified phase is reached, which can result in prolonged treatment times. The DTT technique addresses these challenges by automatically adjusting the irradiation position according to the target’s respiratory motion, enabling continuous, tumor-focused beam delivery without requiring breath-holding and potentially reducing the treatment time.

Factors reducing the accuracy of DTT in stereotactic body radiotherapy (DTT-SBRT) include the accuracy of the correlation model between the tumor and external respiratory signals [6], [7], [8], mechanical accuracy [9], and target localization error (TLE) [10], [11], [12], [13], [14], [15].

In DTT-SBRT, fiducial markers are typically implanted near the tumor to serve as surrogates because directly visualizing the tumor on fluoroscopic images is challenging. However, the positions of these markers depend on the technique and skill of the operator, and the number of markers used varies among institutions. Consequently, tumors and fiducial markers do not always exhibit synchronous motion across different respiratory phases, in a phenomenon called the “TLE” in this study. To prevent a decrease in the tracking accuracy owing to the TLE, determining an appropriate target volume that compensates for it is essential. At our institution, during the treatment planning for DTT-SBRT, we selected a specific respiratory phase from four-dimensional computed tomography (4D-CT) as the reference phase and determined the target volume in that phase, considering the TLE [11]. However, this method requires the synthesis of respiratory phase images using dedicated tools, which is time-consuming. Previous studies on the TLE assumed a constant relative position of fiducial markers or focused on the importance of marker selection rather than addressing the respiratory-induced asynchronous movement between the tumor and fiducial markers [12], [13], [14], [15].

The purpose of this study was to investigate the TLE caused by asynchronous movement between lung tumors and fiducial markers using 4D-CT. We determined the appropriate margin needed to compensate for these errors and improve the tracking accuracy by identifying an optimal target volume.

## 2 Materials and methods

### 2.1 Patients

This study included data from 21 patients with spherical fiducial markers implanted around their lung tumors. Data from 11 patients were used to determine the TLE, and data from the remaining 10 patients served as the validation dataset (Table 1).

**Table 1:** Patient characteristics. *Abbreviations*: TLE, target localization error; M, male; F, female; L, left; R, right; 4D-CT, four-dimensional computed tomography.

	Cohort for determining TLE (*n* = 11)	Validation cohort (*n* = 10)
Age [Median (range)] (yr)	78 (64–88)	82 (71–90)
Sex (M / F)	9 / 2	7 / 3
Stage (T1a / T1b / T2a / T4 / Meta)	4 / 2 / 3 / 1 / 1	2 / 4 / 2 / 1 / 1
Tumor size [Median (range)] (mm)	20 (10–35)	24 (12–40)
Tumor location (L / R)	3 / 8	2 / 8
Number of fiducial markers at 4D-CT imaging (2 / 3 / 4 / 5)	1 / 2 / 4 / 4	2 / 5 / 3 / 0

In the TLE evaluation cohort, 2–5 fiducial markers, each with a diameter of 1.5 mm, were implanted near the tumor. 4D-CT imaging was performed using an Aquilion ONE scanner (Canon Medical Systems, Tochigi, Japan) equipped with a 320-detector row configuration. The imaging parameters for 4D-CT used to calculate the TLE were a tube voltage of 120 kV and a slice thickness of 1 mm. 320-row 4D-CT scans were used to determine the TLE because the artifacts observed were relatively minor [16], and the TLE could be accurately evaluated. Conversely, the 4D-CT imaging conditions for the validation cohort included a tube voltage of 120 kV. For six patients, a LightSpeed RT scanner (General Electric Medical Systems, Waukesha, WI, USA) with a 16-detector row configuration and a slice thickness of 2.5 mm was used. For the remaining four patients, a SOMATOM Definition AS Open scanner (Siemens Healthineers, Tokyo, Japan) with a 64-detector row configuration and a slice thickness of 2 mm was used.

This study was approved by the Medical Ethics Committee of the Graduate School of Medicine and Faculty of Medicine, Kyoto University, and conducted with permission from the head of the research institution (R1446-2).

### 2.2 Calculation of target localization error

**Fig. 1:**
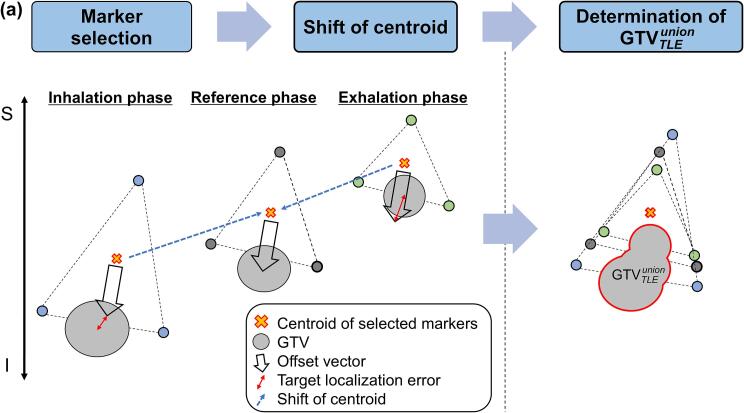

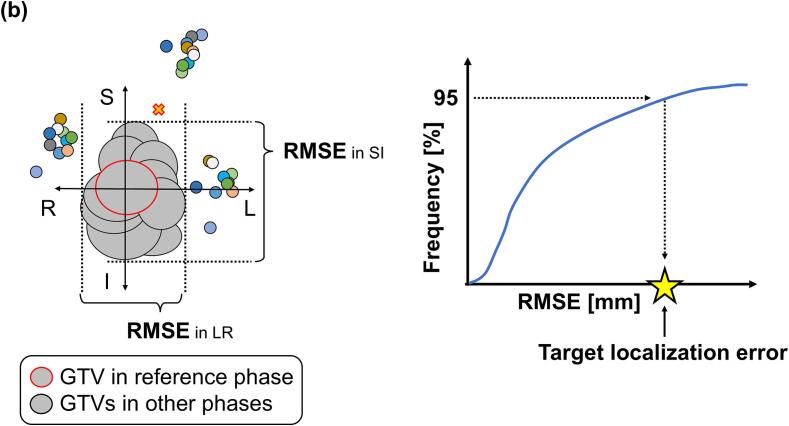
Analysis procedure for cases with four fiducial markers where three markers are used. (a) Workflow for determining the target volume that compensates for the TLE in DTT-SBRT. (b) Method for calculating the RMSE of the GTV centroid in each phase and determining the TLE. The same applies to the AP direction. *Abbreviations*: TLE, target localization error; DTT-SBRT, dynamic tumor-tracking stereotactic body radiotherapy; RMSE, root mean square error; GTV, gross tumor volume; AP, anterior − posterior.

Fiducial marker implantation depends on the operator’s technique and expertise, and the number of markers used may vary across institutions. Therefore, all possible combinations of fiducial markers were evaluated for each case to account for these variations (for example, for cases with five markers, ten distinct patterns were considered when selecting two markers (_5_C_2_ = 10 combinations)). First, the 4D-CT images were divided into 10 respiratory phases, and one radiation oncologist delineated the gross tumor volume (GTV) and measured the marker positions for each phase. The delineated GTVs were used in another study [16]. Next, arbitrary fiducial markers were selected, and the centroids of the fiducial markers in each phase were calculated. Afterward, shifted CT images were created where the centroids of the fiducial markers in the reference phase were aligned with the centroids of the fiducial markers in each phase using MIM Maestro (MIM Software Inc., Cleveland, USA). The 30 % respiratory phase of the 4D-CT was used as the reference phase. Subsequently, the union of the GTVs across all respiratory phases on the shifted CT images was defined as the ${GTV}_{union}^{shift}$ that compensated for respiratory-induced mismatches between the lung tumor and fiducial marker positions in DTT-SBRT (Fig. 1a).

Additionally, the centroid of the GTV in the reference phase was set as the origin, and the variations in the GTV centroids across the respiratory phases were calculated in three directions: left − right (LR), anterior − posterior (AP), and superior − inferior (SI) using the root mean square error (RMSE). The 95th percentile value of the RMSE was defined as the TLE (Fig. 1b) as follows:

(1) $$TLE=P_{95}\left(RMSE\right)=P_{95}\left(\sqrt{\frac{1}{N}\sum_{i=1}^{N}{\left(G_{i}-{\hat{G}}_{i}\right)}^{2}}\right),$$

where *P*_95_(RMSE) denotes the 95th percentile value of the RMSE. The 95th percentile value was adopted to exclude artifacts that could affect GTV delineation [17]. *N* represents the number of respiratory phases excluding the reference phase, which is 9. $G_{i}$ denotes the GTV centroids across the respiratory phases other than the reference phase, and ${\hat{G}}_{i}$ signifies the GTV centroids in the reference phase.

Furthermore, the standard deviation (SD) of RMSE was calculated as an estimate of uncertainty.

### 2.3 Correlation analysis of TLE

**Fig. 2:**
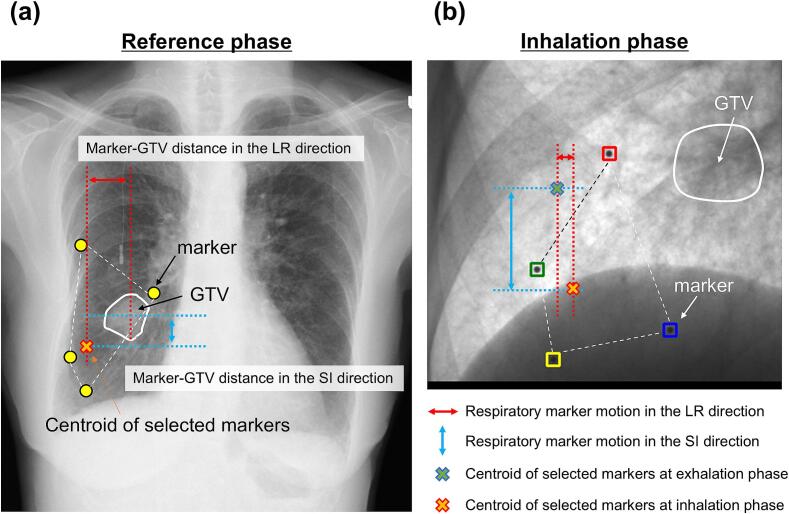
Definitions of the marker–GTV distance and respiratory marker motion. (a) Reference phase and (b) Inhalation phase. The same applies to the AP direction. *Abbreviations*: GTV, gross tumor volume; AP, anterior − posterior.

For the TLE calculation cohort (Table 1), Pearson correlation analyses were conducted to assess the relationship between the TLE and the distance between the centroid of the selected fiducial marker and the centroid of the GTV in the reference phase (marker–GTV distance), as well as the maximum distance moved by the centroid of the selected fiducial marker across all respiratory phases (respiratory marker motion). Fig. 2 presents the definitions of the marker–GTV distance and respiratory marker motion.

### 2.4 Validation

In each case, the GTV with the TLE calculated from all combinations (${GTV}_{TLE}^{ref}$) was compared with the ${GTV}_{union}^{shift}$ in the reference phase. The evaluation metrics included the volume ratio of ${GTV}_{TLE}^{ref}$ to ${GTV}_{TLE}^{union}$ (${rV}_{union}^{ref}$) and the marker–GTV distance.

Furthermore, an isotropic 5 mm margin was added to ${GTV}_{TLE}^{ref}$ to create the planning target volume (${PTV}_{TLE}^{ref}$). We defined the clinical target volume (CTV) as equivalent to the GTV. A DTT-SBRT plan was created using coplanar VMAT with two half-arcs in the reference phase of the 4D-CT, and the dose distribution was evaluated. RayStation ver. 2023B (RaySearch Laboratories, Stockholm, Sweden) was used for treatment planning, and the collapsed cone algorithm was employed for dose calculation. The calculation grid size was set to 2 mm. The treatment machine selected was OXRAY (Hitachi, Ltd., Tokyo, Japan) [18]. The prescribed dose was 50 Gy in four fractions, with the ${PTV}_{TLE}^{ref}$ covering 95 % of the dose (D_2%_: 133–143 %) [19].

## 3 Results

Table 2 presents the results of the TLE for the GTV based on the number of fiducial markers selected. Considering all possible combinations of two or more fiducial markers in each case, the total number of combinations per direction was 157 (71 combinations for two markers, 58 for three markers, 24 for four markers, and 4 for five markers). The TLE decreased as the number of markers increased. When considering all combinations, the TLEs (SD of the RMSE) were 2.0 (0.8) mm, 2.1 (0.7) mm, and 3.2 (1.1) mm in the LR, AP, and SI directions, respectively.

**Table 2:** Ranges of the root mean square error (RMSE) and target localization error (TLE). The 95th percentile value of the RMSE is defined as the TLE. *Abbreviations*: LR, left − right; AP, anterior − posterior; SI, superior − inferior; SD, standard deviation.

No. of fiducial markers selected	LR [mm]		AP [mm]		SI [mm]	
	RMSE (SD)	TLE	RMSE (SD)	TLE	RMSE (SD)	TLE
Two (*n* = 71)	0.2 − 4.9 (0.3)	2.2	0.2 − 4.3 (0.5)	2.8	0.2 − 7.1 (0.6)	4.0
Three (*n* = 58)	0.2 − 3.9 (1.1)	2.0	0.2 − 2.9 (0.8)	2.1	0.4 − 5.3 (1.5)	2.5
Four (*n* = 24)	0.2 − 3.0 (0.4)	0.9	0.2 − 2.0 (0.6)	1.4	0.5 − 4.1 (0.5)	1.4
Five (*n* = 4)	0.2 − 0.7 (0.2)	0.7	0.2 − 1.0 (0.2)	0.9	0.6 − 1.1 (0.3)	1.1
All (*n* = 157)	0.2 − 4.9 (0.8)	2.0	0.2 − 4.3 (0.7)	2.1	0.2 − 7.1 (1.1)	3.2

Supplementary Table S1 summarizes the results of the correlation analysis between the TLE and marker–GTV distance or respiratory marker motion based on the number of markers selected. The correlation coefficient for respiratory marker motion was higher than that for the marker–GTV distance. The strongest correlation was observed in the AP direction. Supplementary Fig. S1 presents the scatter plots of the TLE versus marker–GTV distance and respiratory marker motion.

The mean ± SD values for ${GTV}_{union}^{shift}$ and ${GTV}_{TLE}^{ref}$ were 22.6 ± 15.7 cm^3^ (range: 2.1–45.1 cm^3^) and 19.4 ± 12.7 cm^3^ (range: 1.9–39.9 cm^3^), respectively. Comparing ${GTV}_{union}^{shift}$ and ${GTV}_{TLE}^{ref}$, the mean ± SD values for ${rV}_{union}^{ref}$ and the centroid distance were 1.0 ± 0.2 (range: 0.7–1.5) and 2.7 ± 1.5 mm (range: 0.0–5.0 mm), respectively. Supplementary Fig. S2 presents the cross-sectional views of the case with the smallest ${GTV}_{union}^{shift}$.

**Fig. 3:**
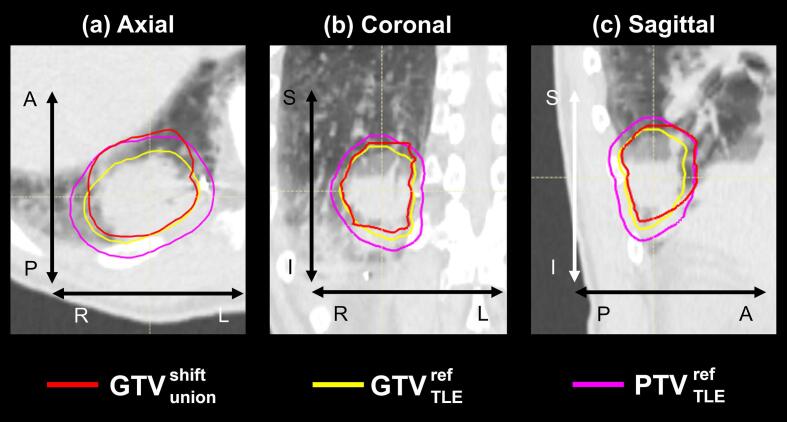
${GTV}_{union}^{shift}$ (red), ${GTV}_{TLE}^{ref}$ (yellow), and ${PTV}_{TLE}^{ref}$ (purple) for the case with the lowest ${GTV}_{union}^{shift}$ V_100%_ at the isocenter level. *Abbreviations*: GTV, gross tumor volume; ref, reference phase (see Fig. 1a); V_100%_, the percent volume of the region of interest receiving 100% of the prescribed dose. (For interpretation of the references to colour in this figure legend, the reader is referred to the web version of this article.)

**Fig. 4:**
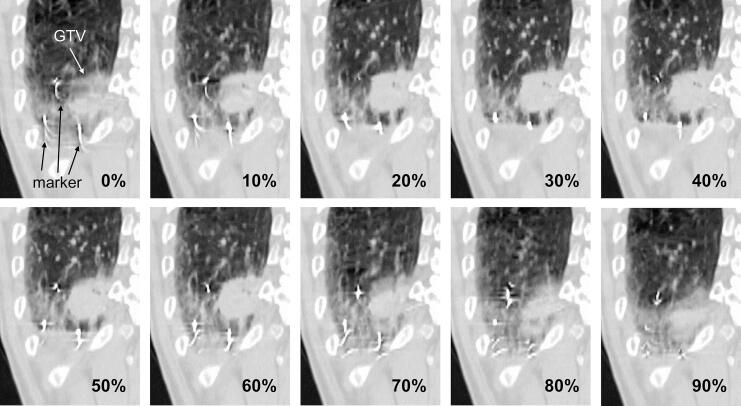
4D-CT images of the case with the lowest ${GTV}_{union}^{shift}$ V_100%_. Spherical fiducial markers with a diameter of 2 mm appear approximately 10 mm in length or are divided into several parts owing to severe artifacts. *Abbreviations*: 4D-CT, four-dimensional computed tomography; GTV, gross tumor volume.

The evaluation of V_100%_ (the percent volume of the region of interest receiving 100 % of the prescribed dose) for ${GTV}_{TLE}^{ref}$ and ${GTV}_{union}^{shift}$ revealed that V_100%_ was 100 % for all cases of ${GTV}_{TLE}^{ref}$, whereas V_100%_ for ${GTV}_{union}^{shift}$ ranged from 97.0 % to 100 %. Supplementary Table S2 presents the characteristics of the cases with ${GTV}_{union}^{shift}$ V_100%_ below 100 %. Fig. 3 illustrates the cross-sectional views of ${GTV}_{union}^{shift}$, ${GTV}_{TLE}^{ref}$, and ${PTV}_{TLE}^{ref}$ for the case with the lowest V_100%_ of ${GTV}_{union}^{shift}$. A portion of ${GTV}_{union}^{shift}$ was not included in ${PTV}_{TLE}^{ref}$; this case was characterized by significant artifacts (Fig. 4). The spherical fiducial markers with a diameter of 2 mm appeared approximately 10 mm in length owing to artifacts. Similar trends were observed for the other three cases.

## 4 Discussion

We estimated the margins needed to compensate for asynchronous movement between lung tumors and fiducial markers due to respiration using 4D-CT. This estimation is critical for DTT-SBRT, where beams are delivered across all respiratory phases. The results indicated that anisotropic margins are essential to account for the TLE of the GTV in the reference phase. Furthermore, validation revealed that applying anisotropic margins maintained a high V_100%_, although artifact-related discrepancies impacted a few cases.

We calculated the TLE by considering all possible combinations using two or more fiducial markers. The TLE decreased with increasing number of markers, suggesting that the markers were implanted to encircle the tumor. Ueki et al. demonstrated that, when assuming constant relative positions of fiducial markers, variations in the distance are particularly pronounced in the SI direction [13], supporting the findings of this study.

Pearson correlation analyses were conducted between the TLE and the marker–GTV distance, as well as the respiratory marker motion. The respiratory marker motion had a stronger influence than the marker–GTV distance in all directions, with the highest correlation coefficients observed in the AP direction for the marker–GTV distance and respiratory marker motion (Supplementary Table S1). This suggests minimal movement of the fiducial marker centroid, possibly because of the position of the markers and the effects of lung expansion and contraction. Conversely, the absence of a correlation in the LR direction may be attributed to limited respiratory marker motion and the resulting small TLE. Similarly, in the SI direction, the high correlation between the tumor and markers, alongside the small TLE, likely accounted for the lack of an observed correlation. Smith et al. indicated that the impact of respiratory motion increases when fiducial markers are positioned closer to the diaphragm [20]. Contrarily, other studies have suggested that fiducial markers implanted near the tumor reflect tumor motion more accurately [13], [21]. In this study, the analysis of the marker–GTV distance and TLE revealed a significant correlation only in the AP direction. This finding suggests that the influence of the TLE because of tumor − fiducial marker asynchrony is minimal across various fiducial marker positions, except in the AP direction.

In this study, the validity of the estimated margins was evaluated from geometric and dosimetric perspectives. First, to compare the ${GTV}_{union}^{shift}$ and ${GTV}_{TLE}^{ref}$ on the CT images, we focused on the volume ratio and centroid distance. Comparison of ${GTV}_{TLE}^{ref}$ and ${GTV}_{union}^{shift}$ revealed that ${GTV}_{TLE}^{ref}$ was 10 % smaller in terms of the median, with a centroid distance difference of 2.9 mm. The dose distribution for ${PTV}_{TLE}^{ref}$ was calculated, and the V_100%_ of ${GTV}_{union}^{shift}$ was evaluated. Of the 10 cases, the ${GTV}_{union}^{shift}$ V_100%_ was less than 100 % in four cases (Supplementary Table S2), and insufficient dose coverage was mainly present in the AP direction (Fig. 3); severe artifacts were observed in these cases (Fig. 4). The discrepancy between ${GTV}_{TLE}^{ref}$ and ${GTV}_{union}^{shift}$ was likely because of an incorrect assessment of marker positions caused by artifacts. This suggests that ${GTV}_{union}^{shift}$ determination may also be subject to overestimation in clinical practice.

The validity of the margins was also considered from a clinical perspective. Matsuo et al. conducted a multi-institutional clinical trial on DTT-SBRT for lung cancer and reported a 2-year local control rate of 95.2 % [19]. In their study, all participating institutions, except ours, defined ${GTV}_{union}^{shift}$ by applying a 2 mm margin in all directions to the GTV in the reference phase (private communication). However, selecting a 2 mm margin to determine ${GTV}_{union}^{shift}$ lacks scientific validation. We calculated the TLE using 320-row 4D-CT scans, and this value was used to determine the appropriate margin. Consequently, while the margin estimated in this study was ≥ 2 mm, the calculation of the TLE was considered clinically relevant. When determining the PTV, considering the TLE and other sources of uncertainty, such as correlation model errors, baseline drift, mechanical errors of radiation therapy equipment, and variations in fiducial markers, is critical [19], [22].

In DTT, beams are delivered across all respiratory phases. This study utilized data from all respiratory phases to obtain its results, making the findings applicable to DTT. Although fiducial markers are also used in respiratory-gated radiotherapy, the asynchrony identified in this study could present a potential issue. However, in respiratory-gated radiotherapy, the irradiated phase is limited to a pre-defined respiratory phase rather than encompassing all phases. Consequently, the TLE is expected to be smaller than that observed in this study.

This study had three main limitations. First, the sample size was relatively small, with 11 cases used for TLE evaluation and the remaining 10 cases used for validation. We systematically evaluated all possible combinations of fiducial markers for each case to account for potential variations in the number and positioning of fiducial markers; however, further research involving a larger sample size is necessary to better assess the clinical applicability of margins intended to compensate for TLE. Second, all fiducial markers were used equally in this study, with no exclusions based on their distance from the target. However, including all markers, regardless of their proximity to the tumor, may introduce variability, particularly when different numbers of fiducials are used across patients. Implementing a weighting approach that prioritizes markers based on their distance from the target could provide a more standardized method for data pooling, potentially enhancing the consistency and reliability of our findings. Third, GTV contours may be influenced by various factors. In routine treatment planning, GTV is not typically delineated for each respiratory phase. Instead, only ${GTV}_{union}^{shift}$ is directly determined after various processing steps. However, we analyzed cases in which the GTV was delineated across all respiratory phases. The GTV contours used in this study were delineated by one radiation oncologist, which could have introduced a potential bias. Furthermore, the influence of artifacts on GTV contours at different respiratory phases must be considered. The artifacts observed in the 320-row 4D-CT scans used in this study were relatively minor, as reported by Iizuka et al. [16]. Conversely, 16-row and 64-row 4D-CT scans, used in validation studies, exhibit larger artifacts frequently [17], [23] (Fig. 4). Using a CT scanner with a higher number of detector rows or applying deep learning-based artifact reduction algorithms in CT simulations effectively mitigates the impact of these artifacts on GTV contouring [24], [25].

In conclusion, this study examined the margins required to compensate for respiratory-induced asynchronous movement between lung tumors and fiducial markers using 4D-CT. Using a conventional target volume determination procedure designed to compensate for the TLE in DTT-SBRT, we aligned the centroids of the fiducial markers and calculated the RMSE between the GTV in the reference phase and the GTV in each respiratory phase. This approach allowed us to assess the TLEs and apply appropriate margins. The margins in the LR, AP, and SI directions were 2.0, 2.1, and 3.2 mm, respectively. Additionally, the validation results suggest that the target volume determination method with margins applied to compensate for TLEs relative to the GTV in the reference phase could replace the current target volume determination method without the influence of artifacts.

## Data availability statement

The data are not publicly available because of privacy or ethical restrictions.

## CRediT authorship contribution statement

**Seiya Matsumoto:** Methodology, Formal analysis, Data curation, Investigation, Writing – original draft. **Nobutaka Mukumoto:** Methodology, Data curation, Writing – review & editing. **Tomohiro Ono:** Data curation, Writing – review & editing. **Hiraku Iramina:** Data curation, Writing – review & editing. **Hideaki Hirashima:** Data curation, Writing – review & editing. **Takanori Adachi:** Data curation, Writing – review & editing. **Yuki Miyabe:** Software, Data curation, Writing – review & editing. **Noriko Kishi:** Data curation, Writing – review & editing. **Takashi Mizowaki:** Data curation, Writing – review & editing, Funding acquisition. **Mitsuhiro Nakamura:** Conceptualization, Resources, Data curation, Visualization, Supervision, Project administration, Writing – review & editing, Funding acquisition.

## Funding

This study was supported by a JSPS KAKENHI grant (Grant-in-Aid for Scientific Research (B) 23K24282) and the Japan Agency for Medical Research and Development (AMED) (24ck0106924h0001).

## Declaration of competing interest

The authors declare that they have no known competing financial interests or personal relationships that could have appeared to influence the work reported in this paper.
