# Supplementary material for: Margins to compensate for respiratory-induced mismatches between lung tumor and fiducial marker positions using four-dimensional computed tomography
Source: Phys Imaging Radiat Oncol. 2025 Feb 7;33:100728. doi: 10.1016/j.phro.2025.100728 (PMC11871501; doi:10.1016/j.phro.2025.100728)
Supplement: Supplementary Data 1 [file mmc1.pdf]

Supplementary Figure S1

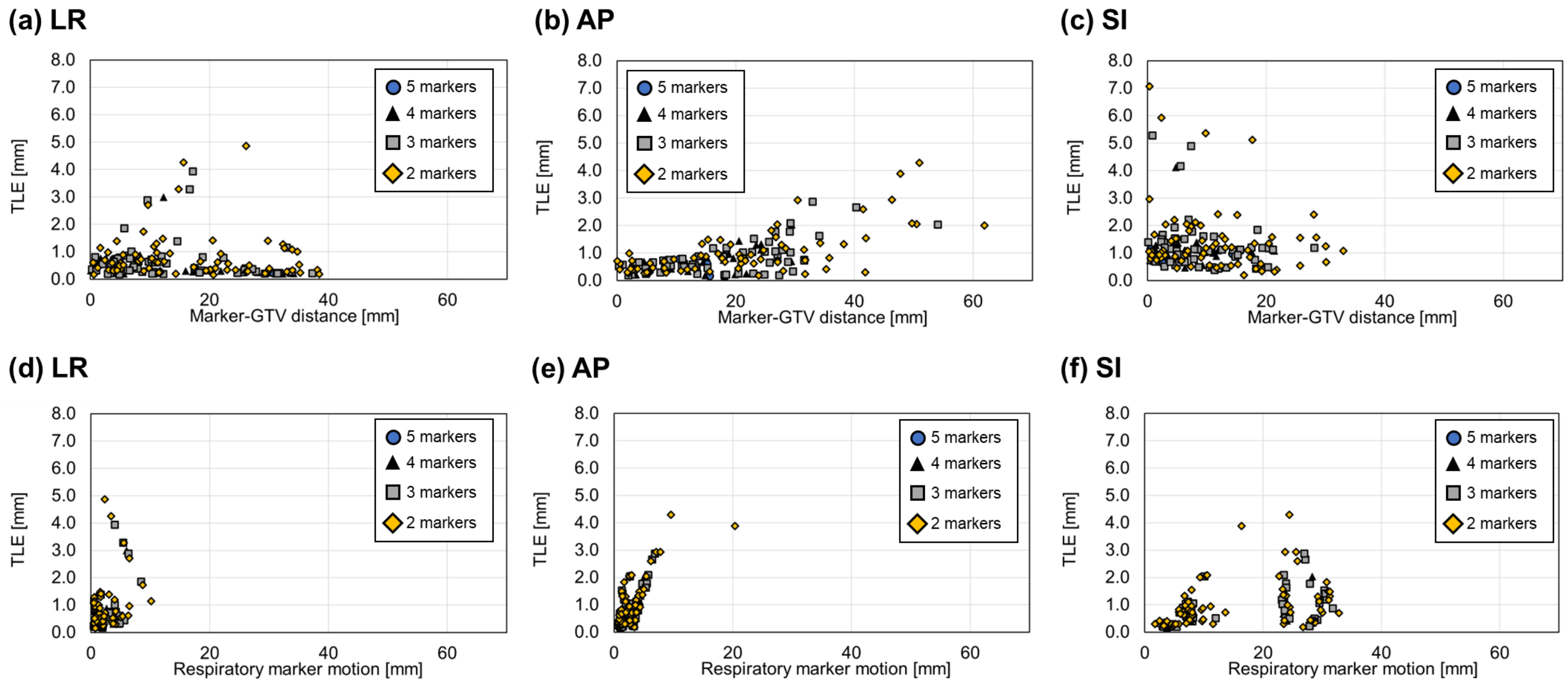

Supplementary Figure S1. Scatter plots of target localization error (TLE) versus marker–GTV distance or respiratory marker motion. (a)–(c) Scatter plots of marker–GTV distance versus TLE; (d)–(f) Scatter plots of respiratory marker motion versus TLE.

Supplementary Figure S2

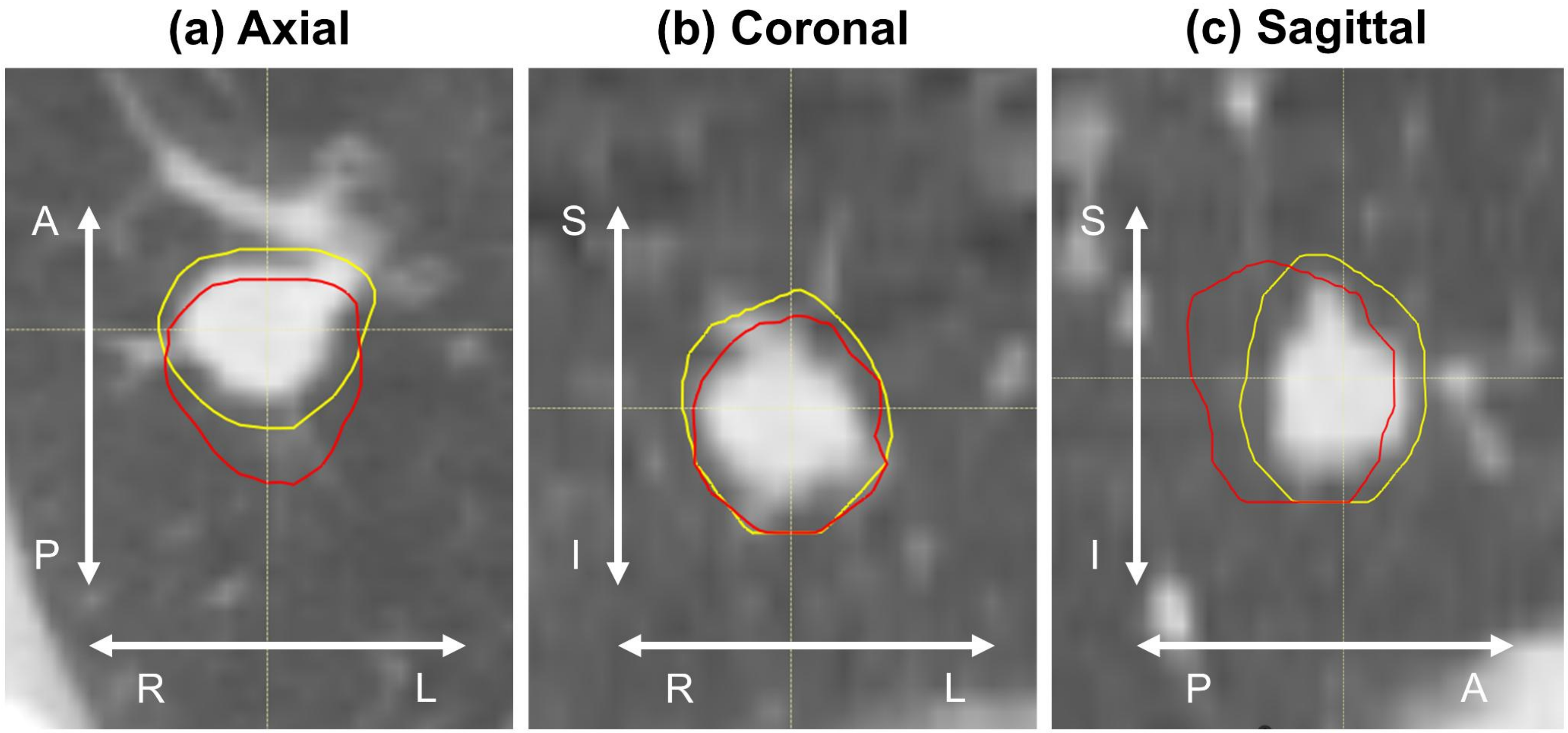

Supplementary Figure S2. GTV_{union}^{shift} (red) and GTV_{TLE}^{ref} (yellow) for the case with the smallest GTV_{union}^{shift} on the isocenter plane. (GTV_{union}^{shift} = 2.1 cm^3, GTV_{TLE}^{ref} = 1.9 cm^3, rV_{union}^{ref} = 0.91, centroid distance = 3.1 mm).

Supplementary Table S1. Pearson correlation coefficients between marker–GTV distance or respiratory marker motion and target localization error (TLE). A positive value indicates a correlation between marker–GTV distance or respiratory marker motion and TLE. *Abbreviations*: LR, left−right; AP, anterior−posterior; SI, superior−inferior.

* Marker–GTV distance: the distance between the centroid of the selected fiducial marker and the centroid of the GTV in the reference phase.

** Respiratory marker motion: the maximum distance that the centroid of the selected fiducial marker moved across all respiratory phases.

	LR		AP		SI	
No. of fiducial markers selected	Distance*	Motion**	Distance*	Motion**	Distance*	Motion**
Two (*n*=71)	0.00	0.31	0.66	0.76	-0.18	0.27
Three (*n*=58)	-0.12	0.51	0.63	0.73	-0.19	0.39
Four (*n*=24)	-0.22	0.58	0.55	0.76	-0.11	0.45
Five (*n*=4)	-0.74	0.14	0.06	0.96	-0.07	0.48
All (*n*=157)	0.05	0.41	0.65	0.76	0.14	0.33

Supplementary Table S2. Characteristics of four cases with GTV_{union}^{shift} $V_{100\%}$ below 100%. *Abbreviations*: $V_{100\%}$, the percent volume of the region of interest receiving 100% of the prescribed dose; LR, left–right; AP, anterior–posterior; SI, superior–inferior; CT, computed tomography.

* Measured on 4D-CT.

** The distance between the furthest point of GTV_{union}^{shift} that was not included within the PTV_{TLE}^{ref} and the edge of the PTV_{TLE}^{ref} on the isocenter plane. Zero means that GTV_{union}^{shift} is included in PTV_{TLE}^{ref}.

GTV_{union}^{shift} $V_{100\%}$ [%]	97.0	97.5	98.2	99.0
Number of markers implanted	3	4	3	2
Number of detectors of CT scanner	64	16	16	16
Respiratory motion of GTV_{union}^{shift} (LR, AP, SI) [mm] *	(1.1, 4.0, 13.8)	(2.5, 3.5, 19.1)	(0.5, 2.9, 8.6)	(0.9, 7.1, 16.1)
Respiratory motion of centroid of markers (LR, AP, SI) [mm] *	(1.3, 3.5, 16.0)	(2.3, 2.5, 14.4)	(0.7, 4.0, 8.3)	(1.0, 6.1, 16.2)
GTV_{union}^{shift} [cm^3] | GTV_{TLE}^{ref} [cm^3]	41.5 | 37.0	13.3 | 10.0	10.9 | 11.4	31.9 | 26.4
Centroid distance between GTV_{union}^{shift} and GTV_{TLE}^{ref} [mm]	5.0	2.8	4.2	1.6
Distance (L, R, A, P, S, I) [mm] **	(0, 0, 1, 0, 0, 0)	(0, 0, 0, 1.6, 1.6, 0)	(0, 0, 0, 1.6, 1.6, 0)	(0, 0, 1, 0, 0, 0)
